# Spontaneous formation of neutrophil extracellular traps is associated with autophagy

**DOI:** 10.1038/s41598-021-03520-4

**Published:** 2021-12-14

**Authors:** Yun Guo, Fei Gao, Xin Wang, Zhenzhen Pan, Qian Wang, Shiyao Xu, Shanshan Pan, Ling Li, Deyu Zhao, Jun Qian

**Affiliations:** 1 Department of Respiratory Medicine, The Affiliated Wuxi Children’s Hospital of Nanjing Medical University, Wuxi, 214023 Jiangsu China; 2 Department of Respiratory Medicine, Children’s Hospital of Nanjing Medical University, Nanjing, 210000 Jiangsu China; 3 Department of Intensive Care Unit, The Affiliated Wuxi People’s Hospital of Nanjing Medical University, Wuxi, China

**Keywords:** Innate immune cells, Lymphocytes

## Abstract

Neutrophils release neutrophil extracellular traps (NETs), via NETosis, as a defense mechanism against pathogens. Neutrophils can release NETs spontaneously; however, the mechanisms underlying spontaneous NETosis remain unclear. Neutrophils isolated from healthy donors were tested for NET formation and autophagy at 1, 6, 12, and 24 h after incubation. Autophagy response was evaluated in response to various autophagy inducers and inhibitors. The relationship between autophagy and NETosis was detected in vivo using an ovalbumin-induced mouse model of asthma. We found that the increase in the proportion of spontaneous NETosis was time-dependent. The number of autophagy-positive cells also increased over time and LC3B protein played an integral role in NET formation. Trehalose (an inducer of mTOR-independent autophagy) treatment significantly increased NET formation, whereas rapamycin (an mTOR-dependent autophagy inducer) did not increase NET release by neutrophils. Compared with the control group, 3-methyladenine (an autophagy sequestration inhibitor) and hydroxychloroquine sulfate (autophagosome-lysosome fusion inhibitor) treatments significantly reduced the percentage of NET-positive cells. In vivo studies on ovalbumin-induced asthma lung sections revealed NETs and LC3B and citH3 proteins were found to co-localize with DNA. Our findings suggest that autophagy plays a crucial role in aging-related spontaneous NETosis.

## Introduction

Neutrophils are the most abundant multifunctional granulocytes and play a key role in the innate immune response. Upon infection, the exogenous products released by pathogens or inflammatory factors released by damaged tissues are detected by neutrophils recruited to the infection site, which then engulf the pathogens by various pathways^1^. Neutrophils perform their antimicrobial functions through an arsenal of mechanisms, such as by engulfing the microbes into a phagosome and its subsequent fusion with intracellular granules upon discharge of the contents to form a phagolysosome. This results in generation of reactive oxygen species (ROS) combined with antimicrobial peptides and release of neutrophil extracellular traps (NETs) to defend against the pathogens^1,2^.

NETs are web-like extracellular chromatin fibers decorated with various antimicrobial proteins, such as myeloperoxidase (MPO), neutrophil elastase, cathepsin G, and high mobility group box-1 protein. NETs can trap and kill various pathogens, including bacteria, viruses, fungi, and parasites, among others^1–6^. However, the release of NETs is a “double-edged sword.” Excess NETs release by neutrophils during lung infection is correlated with lung injury and morbidity^7^. NETs also promote thrombosis in myeloproliferative neoplasms^8^, heparin-induced thrombocytopenia^9^, and Behcet’s disease^10^, among other conditions. NETs have been linked to the pathogenesis of autoimmune diseases, including systemic lupus erythematosus^11^, rheumatoid arthritis^12^, and anti-neutrophil cytoplasmic autoantibody-associated vasculitis^13^. Neutrophils can release NETs spontaneously^14^, and unusual spontaneous release of NETs may trigger various diseases. However, the mechanisms of spontaneous NETosis remain unclear.

Autophagy, also known as type II programmed cell death, differs from apoptosis, and necrosis^15^. It plays an important role in regulating the survival and death of cells and involves the degradation of damaged, degenerated, or aged macromolecular substances within cells using lysosomes as well as self-digestion of damaged organelles^16,17^. Autophagy is associated with NET formation in neutrophils. LC3B-I to LC3B-II conversion, an important marker of autophagy activation, was detected by fluorescence microscopy and immunoblotting during NETosis^11,17,18^. Upon phorbol 12-myristate 13-acetate and lipopolysaccharide stimulation, autophagosomes containing cytosolic components were observed in neutrophils by transmission electron microscopy^18,19^. The association between NETosis and autophagy was also confirmed using various autophagy inducers and inhibitors, which revealed that autophagy inducers improve the release of NETs in response to various stimulations, whereas autophagy inhibitors abrogate tis effect^18–20^.

In this study, we hypothesized that autophagy was involved in the mechanism of spontaneous NETosis. We explored the mechanism of spontaneous NETosis, thereby adding to the existing knowledge available on this phenomenon.

## Methods

### Ethics declarations

The animal experiments were conducted in accordance with Laboratory Animals-Guideline of welfare and ethics and in compliance with the ARRIVE guidelines. The animal experiments was approved by the Institutional Animal Care and Use Committee, Nanjing Medical University (reference number: IACUC-1905058). Human experiments was conducted in accordance with the amended Declaration of Helsinki. Ethical approval was approved by the Ethics Committee of Wuxi Children’s Hospital (WXHC2020-03-003) Informed consent was obtained from all individual participants included in the study and participant under 16 years had a written informed consent obtained from a parent. A total of 24 healthy donors were enrolled in this study, 38.1% of whom were male and the mean age of the children were 11.74 ± 0.49 years.

### Isolation of peripheral blood neutrophils

Neutrophils were isolated from freshly collected blood samples of healthy donors using the Neutrophil Isolation Kit (TBD, Tianjing, China, catalog # LZS11131). Briefly, the freshly collected blood samples from healthy donors were added to the upper layer of the neutrophil isolation reagent and centrifuged for 25 min at 750×g, followed by collection of the neutrophil-enriched supernatant. Residual red blood cells were lysed twice, followed by centrifugation for 10 min at 450×g. Neutrophils were washed twice with PBS and cultured in RPMI 1640 medium supplemented with 10% fatal bovine serum (Biological Industries, Kibbutz Beit Haemek, Israel, catalog # 04-001-1A), for use in subsequent experiments.

### RNA sequencing analysis

The total RNA of neutrophils was extracted from cells using TRIzol reagent (Invitrogen, Waltham, USA, catalog # 15596018) at 1 h for the control group or 6 h for the test group (n = 3) as per the manufacturer’s instructions. RNA purity was checked using the KaiaoK5500 Spectrophotometer (Kaiao, Beijing, China). RNA integrity and concentration was assessed using the RNA Nano 6000 Assay Kit and the Bioanalyzer 2100 system (Agilent Technologies, CA, USA). Library construction and sequencing were performed by Annoroad Gene Technology (Beijing, China). A total amount of 2 μg RNA per sample was used as the input material for RNA sample preparation. Sequencing libraries were generated using NEBNext Ultra RNA Library Prep Kit from Illumina (#E7530L, NEB, USA) according to the manufacturer’s recommendations, and index codes were added to attribute sequences of each sample. The clustering of the index-coded samples was performed on a cBot cluster generation system, using HiSeq PE Cluster Kit v4-cBot-HS (Illumina) according to the manufacturer’s instructions. After cluster generation, the libraries were sequenced on the Illumina Nova seq6000 platform and 150-bp paired-end reads were generated. Sprofile was used to analyze the differential alternative splicing events.

### Cell immunofluorescence

Neutrophils (5 × 10^5^ cells mL^−1^), in the RPMI 1640 medium with 2% FBS, were incubated in confocal dishes for 1, 6, 12, and 24 h. In the autophagy intervention group, neutrophils were primed with either rapamycin (Rap, 100 nM, Selleck Chemicals, Texas, USA, catalog # S1039), trehalose (Tre, 10 mM, Sigma, Munich, Germany catalog # T9531), 3-methyladenine (3-MA, 10 μm, Selleck Chemicals, Texas, USA, catalog # S2767), hydroxychloroquine sulfate (HCQ, 10 μm, Selleck Chemicals, Texas, USA, catalog # S4430), or bafilomycin (Baf, 10 μm, Selleck Chemicals, Texas, USA, catalog # S1413) for 1 h and then cultured in RPMI 1640 medium with 2% FBS for 6 h. Subsequently, the cells were fixed in 4% paraformaldehyde for 20 min; the supernatant was removed gently, and the cells were washed twice with PBS. Cells were permeabilized with 0.3% triton X for 10 min and blocked with 10% goat serum (Biological Industries, Kibbutz Beit Haemek, Israel, catalog # 04-009-1B), for 1 h, followed by incubation with anti-MPO mouse monoclonal antibody (mAB) (abcam, Cambridge, UK, catalog #ab25989, 1:100), LC3B mouse mAB (Cell Signaling Technology, Beverly, USA, catalog # 83506, 1:100), and citH3 rabbit mAB (abcam, Cambridge, UK, catalog # ab5103, 1:100) overnight at 4 °C. The cells were washed with PBS and incubated with goat anti-rabbit Alexa Fluor 568 antibody (Thermo Fisher Scientific, Waltham, USA, catalog # A-11011, 1:1000) and goat anti-mouse Alexa Fluor 488 antibody (Cell Signaling Technology, Beverly, USA, catalog # 4408 s, 1:500) for 1 h at 37 °C. Cells were then incubated with DAPI for 10 min and exposed to ProLong Gold Antifade Reagent (Cell Signaling Technology, Beverly, USA, catalog # 4083S and catalog # 9071S, respectively). Cells were visualized using a confocal TCS SP8 microscope (Leica Microsystems, Germany).

### Quantification of NETs

The double-stranded DNA was detected using PicoGreen reagent (Thermo Fisher Scientific, Waltham, USA, catalog # P7589) according to the manufacturer’s instructions. A total of 100 μL cell suspension (1 × 10^6^ cells mL^−1^ in RPMI 1640 medium) was transferred to 96-well plates and incubated for 1, 6, 12, and 24 h. The supernatant was removed gently and replenished with 100 μL PBS; 100 μL 1X Quant-iT PicoGreen reagent was added to the 96-well plates, followed by incubation for 5 min at room temperature. Fluorescence was detected at an excitation wavelength of 502 nm and an emission wavelength of 523 nm using a spectrofluorometer (SpectraMax M2; Molecular Devices, Biberach an der Riß, Germany).

### Werstern blot analysis of autophagy associated proteins

Western blot analysis was used to quantify autophagy associated protein as previous described^21^. In brief, 20 μL of extracted protein was electrophoresed on 12% SDS–polyacrylamide gel electrophoresis, transferred onto nitrocellulose membrane, blocked with the Western blocking buffer and incubated with autophagy associated proteins (LC3B mouse mAB (Cell Signaling Technology, Beverly, USA, catalog # 83506, 1:1000), p62 mouse mAB (abcam, Cambridge, UK, catalog # ab56416, 1:1000), P-4EBP-1 rabbit mAB (abcam, Cambridge, UK, catalog # ab75767, 1:1000), 4EBP-1 rabbit mAB (abcam, Cambridge, UK, catalog # ab32130, 1:1000), P-P70SK rabbit mAB (abcam, Cambridge, UK, catalog # ab109393, 1:1000), P70SK rabbit mAB (abcam, Cambridge, UK, catalog # ab184551, 1:10,000) overnight at 4 °C. These membranes were washed with PBS and incubated with anti-mouse IgG or anti-rabbit IgG conjugated to horseradish peroxidase.

### Animals

Female BALB/c mice, aged 6–8 weeks, were purchased from the animal care facility of Nanjing Medical University (Jiangsu, China). Animals were kept in a temperature-controlled room (22–23 °C) under a 12 h light/dark cycle, in the laboratory animal house of Nanjing Medical University, and had access to food and water ad libitum. All mice were randomly divided into two groups: OVA group and normal control group. Mice were sensitized by intraperitoneal injection with 20 μg OVA with 1 mg aluminum hydroxide, dissolved in 200 μL PBS, at day 1 and 14. On days 21 to 27, mice were challenged with 1% OVA dissolved in 5 mL PBS by intranasal inhalations for 30 min.

### Hematoxylin and eosin (H&E) staining

After the last challenge, mice were euthanized using chloral pentobarbital. The lung samples were collected and fixed in 4% paraformaldehyde and dehydrated using graded alcohol. Subsequently, the tissues were embedded in paraffin and sliced. H&E staining was performed as described previously^22^.

### Tissue immunofluorescence

Tissue immunofluorescence was performed as described previously^23^. Tissue sections were deparaffinized and rehydrated. Sections of the lung tissue were microwaved in the EDTA antigen repair buffer (pH 8.0) for 15 min to recover the antigen. The sections were then permeabilized with 0.3% triton for 10 min and blocked with BSA for 30 min, followed by overnight incubation with antibody against Ly6G mouse mAB (Goodbio technology, Hubei, China, catalog # GB11229, 1:3000) at 4 °C. The sections were washed with PBS and incubated with goat anti-mouse HRP-labeled antibody (Goodbio technology, Hubei, China, catalog # GB23301, 1:500) for 50 min at 37 °C. The sections were again washed with PBS and incubated with CY3 (Goodbio technology, Hubei, China, catalog # G1223) for 5 min. The lung sections were again microwaved in the EDTA antigen repair buffer (pH 8.0) for 15 min to recover the antigen. The sections were then incubated with antibodies against LC3B mouse mAB (Cell Signaling Technology, Beverly, USA, catalog # 83506, 1:100) and citH3 rabbit mAB (abcam, Cambridge, UK, catalog # ab5103, 1:100) overnight at 4 °C. This was followed by washes with PBS and incubation with goat anti-rabbit FITC (Goodbio technology, Hubei, China, catalog # GB22303, 1:300) and goat anti-mouse CY5 antibody (Goodbio technology, Hubei, China, catalog # GB27301) for 50 min at 37 °C. An autofluorescence quenching agent (Goodbio technology, Hubei, China, catalog #, catalog # G1221) was added for 5 min, followed by rinsing with running water for 10 min. The lung sections were then incubated with DAPI for 10 min and exposed to ProLong Gold Antifade Reagent (Cell Signaling Technology, Beverly, USA, catalog # 4083S and catalog # 9071S, respectively). Sections were visualized using a confocal TCS SP8 microscope (Leica Microsystems, Germany).

### Statistical analysis

SPSS Statistics 23.0 for Win10 (IBM, New York, U.S.) was used for data processing. Normally distributed data were expressed as mean ± standard deviation. One-way ANOVA used to analyze the differences between groups. For multiple comparison tests, Dunnett's test was select if one column represents control data and Tukey test was chosen if compare all pairs of columns. A *P*-value < 0.05 was considered statistically significant.

## Results

### Aging-related spontaneous NETosis

**Figure 1:**
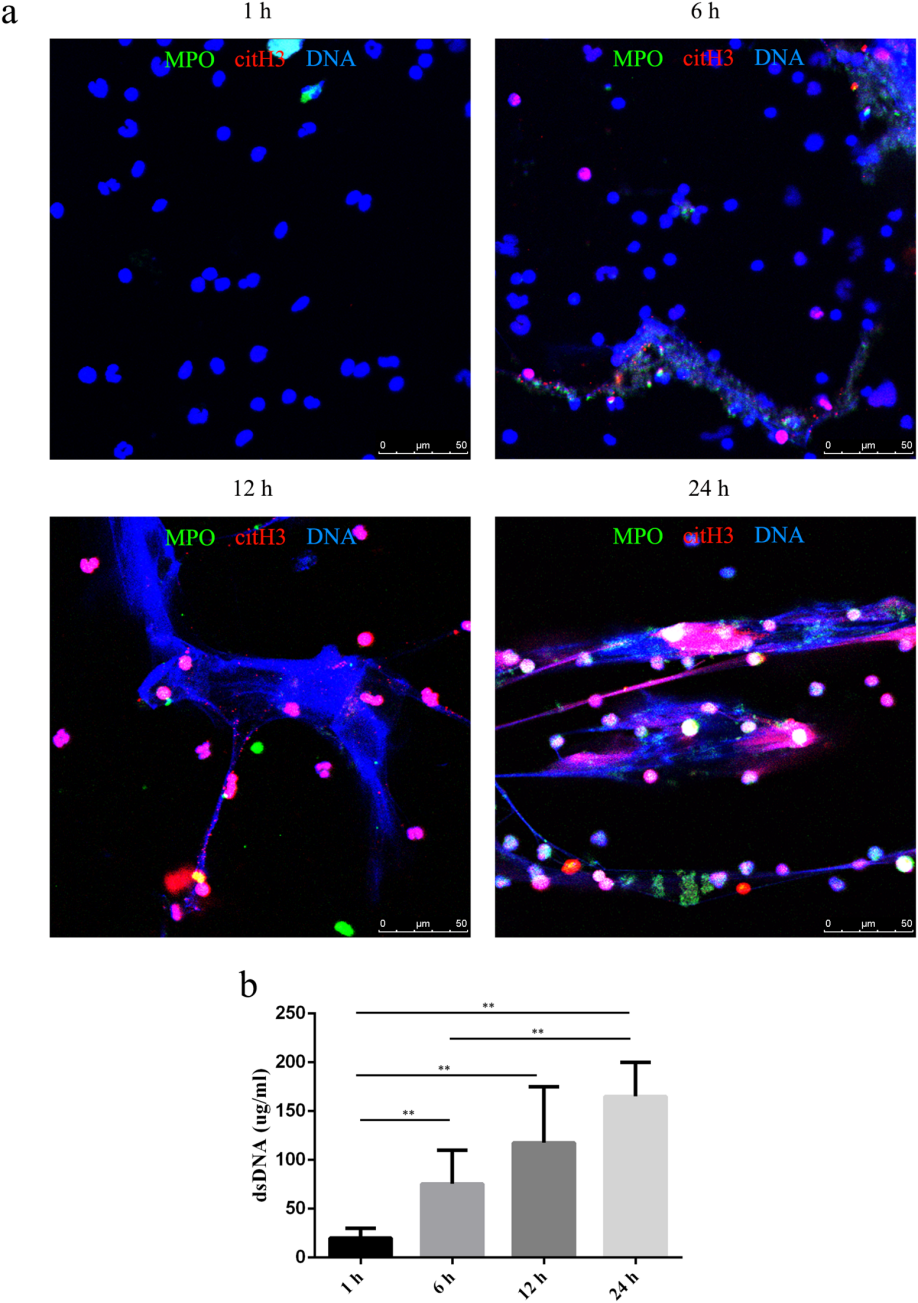
Aging-related spontaneous NETosis. The proportion of spontaneous NETs and dsDNA released by neutrophils was evaluated by fluorescence microscopy (**a**) and PicoGreen staining (**b**) at 1, 6, 12, and 24 h. (**a**) Samples were stained with antibodies against citrullinated histone H3 (citH3, red) and myeloperoxidase (MPO, green) and DNA was counterstained with DAPI (blue). Original magnification, 40× ; scale bar, 50 μm. (**b**) DNA release was compared between different groups, and the data are represented as the median with range from six independent experiments, **P < 0.01.

Neutrophils can be stimulated to form NETs in response to numerous pathogens and different signals^1^, and they can also form spontaneously^14^. Given the short half-life of neutrophils^24^, we hypothesized that the spontaneous formation of NETs was time-dependent. To investigate various time-points and efficiency of spontaneous NETosis, we tested the proportion of spontaneous NET formation after 1, 6, 12, and 24 h of incubation. Very low to no spontaneous NETosis was observed at 1 h (Fig. 1a, Supplementary Fig. 1). Over time, spontaneous release of NETs by neutrophils was visualized by fluorescent microscopy (Fig. 1a, Supplementary Fig. 1). Quantification of NETs by PicoGreen showed that DNA release by neutrophils increased in a time-related manner (Fig. 1b).

### RNA-seq analysis in spontaneous NETosis

**Figure 2:**
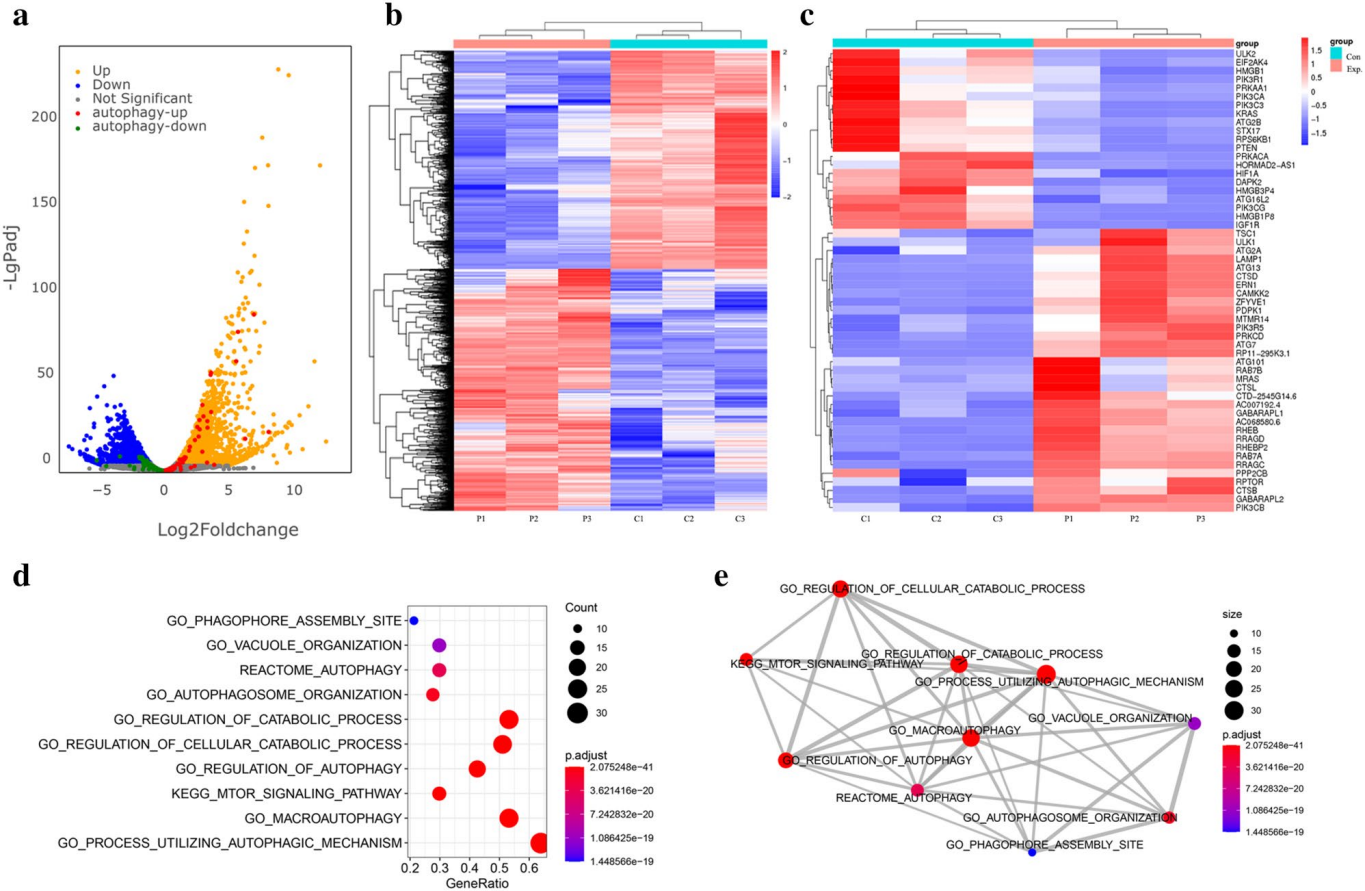
RNA-seq analysis in spontaneous NETosis. Neutrophils from three healthy control children were isolated and divided into two groups, which were further divided into 1 and 6 h groups. Total RNA of cells was harvested at 1 h (control group) and 6 h (spontaneous NETosis group) and RNA-seq data was analyzed as described in the Methods. (**a**) Volcano map of differentially expressed genes between aging neutrophils and controls. Values are presented as log2. Up- and down-regulated genes are colored in orange and blue, respectively. Autophagy-up and -down related genes are colored in red and green, respectively. Hierarchical clustering with heat map showing the RNA levels of differentially expressed (**b**) genes associated with autophagy and (**c**) genes in aging neutrophils and controls. Genes are ranked based on their fold-change. (**d**, **e**) Gene set enrichment scatter plot and overlapping gene set network plot showed differentially expressed genes enriched in autophagy pathways.

As described above, significant release of spontaneous NETs was observed at 6 h compared to at 1 h. Considering that the half-life of neutrophils is less than 7 h^24^, we used the 6 h time-point to evaluate spontaneous NETosis in neutrophils. To investigate the signaling pathway mediating aging-associated spontaneous NETosis by neutrophils, we explored the differential RNA expression by performing RNA sequencing in aging neutrophils (6 h) and control neutrophils (1 h) (n = 3). Results of RNA sequencing revealed a total of 4833 differentially expression genes (2538 with upregulated expression and 2295 with downregulated expression), using the criteria of a fold-change > 2 and *P* < 0.05 (Fig. 2a). Hierarchical clustering with heat maps showed the RNA levels of all differentially expressed genes as well as genes associated with autophagy in aging and control neutrophils (Fig. 2b, c). Gene set enrichment analysis indicated that the differentially expressed genes were enriched in autophagy pathways (Fig. 2d, e).

### Autophagy in spontaneous NETosis

**Figure 3:**
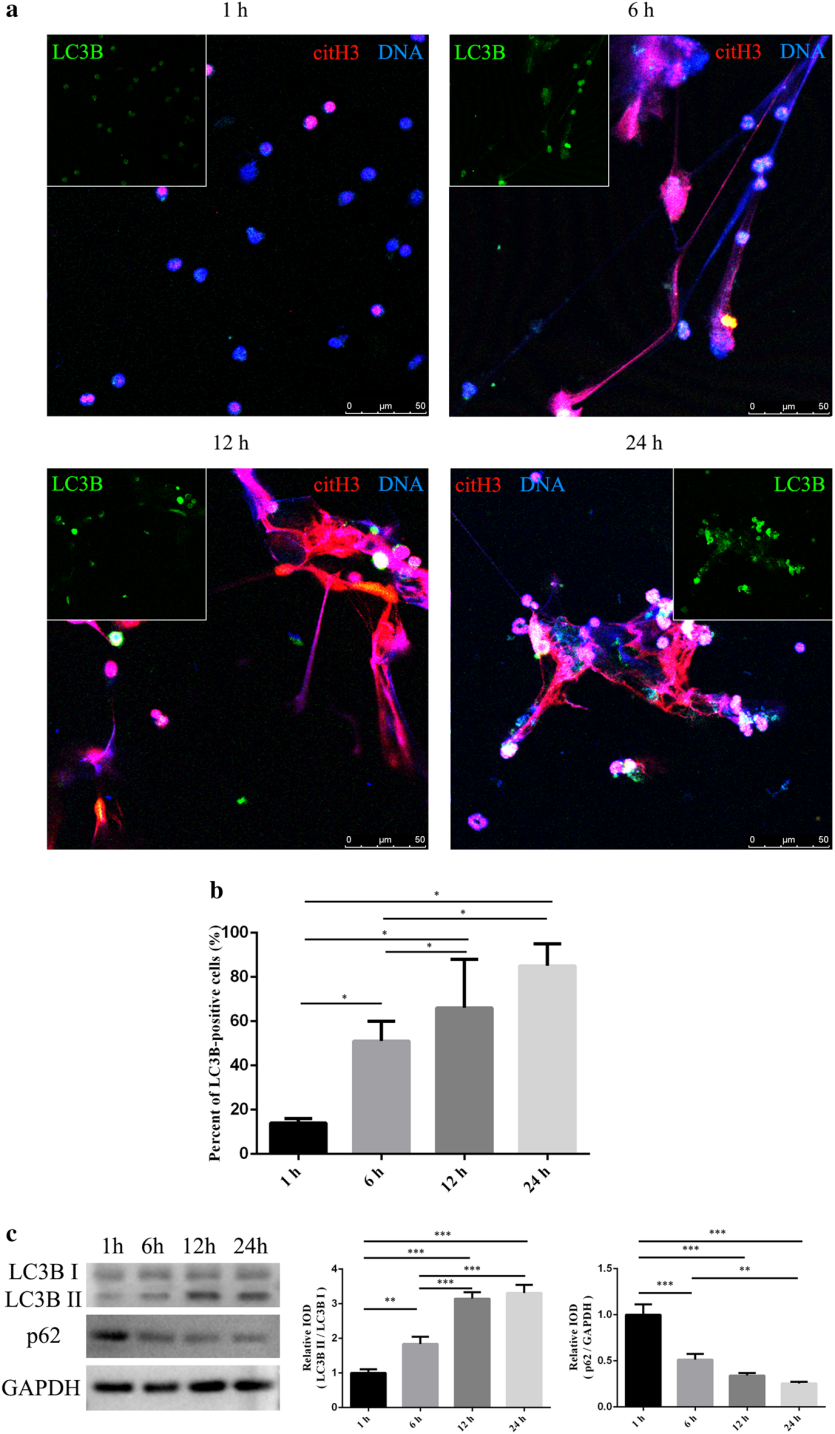
Autophagy in spontaneous NETosis. The percentage of cells exhibiting active autophagy was analyzed by measuring LC3B expression via immunofluorescence and LC3B and p62 expression by immunoblotting after 1, 6, 12, and 24 h of incubation. (**a**) Samples were stained with antibodies specific for citrullinated histone H3 (citH3, red) and light chain 3 B (LC3B, green), and the DNA was counterstained with DAPI (blue). Original magnification 20× ; scale bar, 50 μm. (**b**) Percentage of LC3B-positive cells was determined by counting the number of LC3B-positive cells and total cells in four random fluorescence scanning planes. Data was analyzed from three independent experiments using ImageJ software, *P < 0.05. (**c**) Integrated optical density (IOD) of LC3B II/ LC3B I and p62/GAPDH; data are presented as the mean ± SD of three independent experiments, *P < 0.05, **P < 0.01, ***P < 0.001.

To assess the role of autophagy in NET formation, we investigated the percentage of autophagic cells after 1, 6, 12, and 24 h of incubation by evaluating LC3B expression via immunofluorescence. We examined autophagy levels by immunoblotting for lapidated LC3B and p62 consumption. The results showed that LC3B protein expression was significantly higher at 6 h than that at 1 h and the autophagy level increased over time (Fig. 3a–c, Supplementary Figs. 2, 3, 8). Furthermore, we found that LC3B protein was an integral part of NETs (Fig. 3a, Supplementary Figs. 2, 3), highlighting the importance of autophagy in NET formation.

### Spontaneous NETosis is enhanced by activation of autophagy in neutrophils

**Figure 4:**
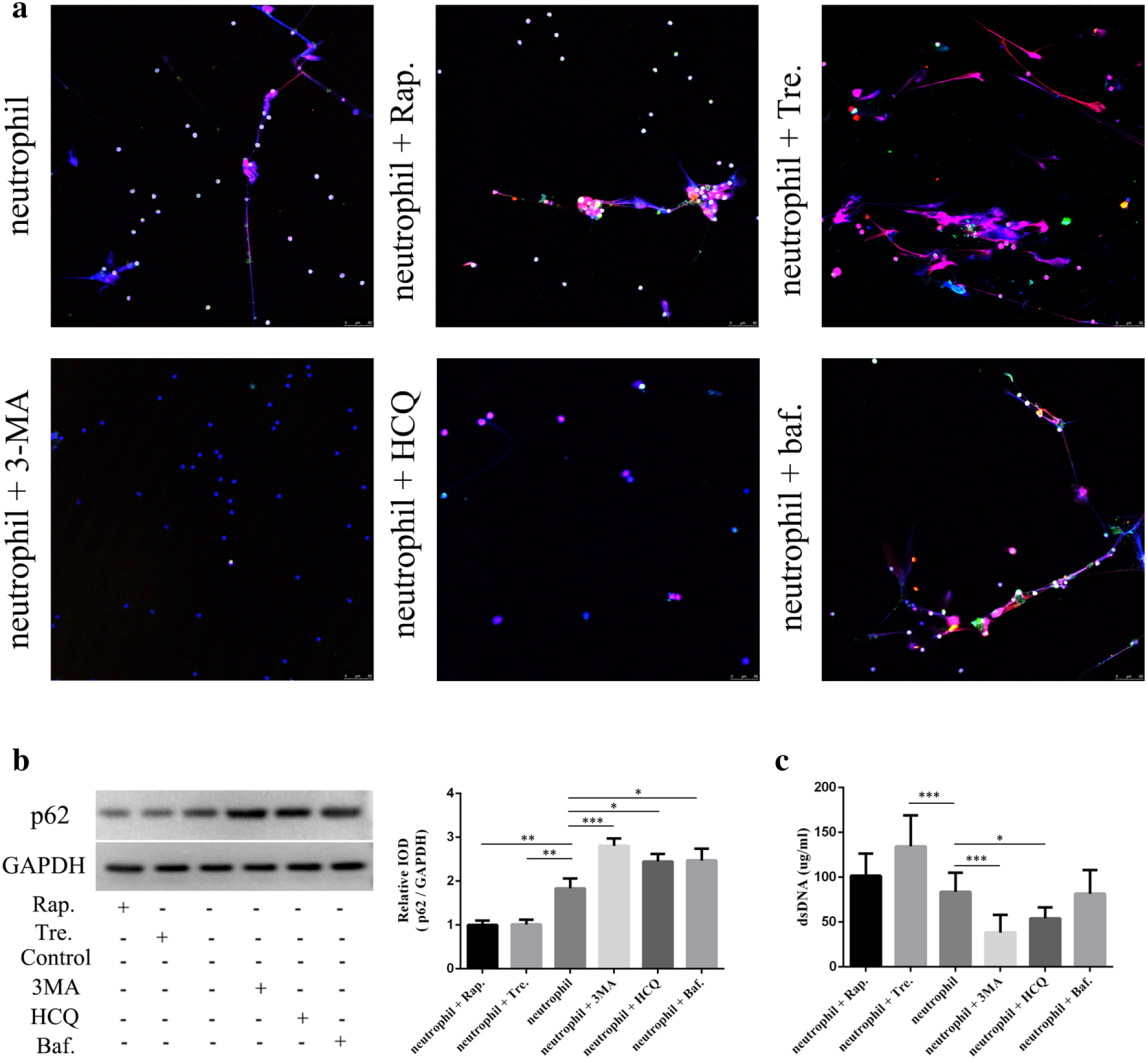
Activation of neutrophil autophagy enhances spontaneous NETosis. Two autophagy inducers rapamycin (Rap.) and trehalose (Tre.) and three autophagy inhibitors 3-methyladenine (3-MA), hydroxychloroquine sulfate (HCQ), and bafilomycin (Baf.) were used to detect the role of autophagy signals in NET formation by fluorescence microscopy (**a**) and PicoGreen staining (**c**) at 6 h. (**a**) Samples were stained with antibodies specific for citrullinated histone H3 (citH3, red) and light chain 3 B (LC3B, green) and DNA was counterstained with DAPI (blue); original magnification 20× ; scale bar, 50 μm. (**b**) Integrated optical density (IOD) of p62/GAPDH, data were presented as the mean ± SD from three independent experiments, *P < 0.05, **P < 0.01, ***P < 0.001. (**c**) DNA release was compared between different groups; data are represented as the median with the range from six independent experiments, *P < 0.05, **P < 0.01, ***P < 0.001.

Mechanistic target of rapamycin (mTOR) is an evolutionarily conserved protein kinase that negatively regulates autophagy^24–27^. However, autophagy can also be activated via mTOR-independent pathways^28^. We first performed RNA-seq analysis in spontaneous NETosis and found that some of mTOR-mediated autophagy-related genes were differentially expressed in aging and control neutrophils (Fig. 2c). Considering that many mTOR pathway-related changes in autophagy do not involve RNA transcription, but are mediated by protein phosphorylation changes, we evaluated the phosphorylation of 4EBP-1 and 70S6K, the direct downstream substrates of mTOR signaling^18^. However, p-4EBP-1 and p-70S6K were not degraded in aging neutrophils (Supplementary Figs. 4, 10). To determine the role of mTOR signaling pathway in spontaneous NETs, we used two autophagy inducers; neutrophils were exposed to either 100 nM Rap, an inducer of mTOR-dependent autophagy, or 10 mM Tre, an inducer of mTOR-independent autophagy, for 6 h. Both Rap and Tre activated autophagy in neutrophils ((Fig. 4b, Supplementary Fig. 9), whereas Tre treatment significantly increased NET formation (Fig. 4a, c), while Rap did not increase NET release by neutrophils (Fig. 4a, c).

Next, we examined whether inhibiting autophagy reduced NET release. To evaluate the effect of autophagy inhibitor, LC3 conversion was analyze in different time points in the presence and absence of autophagy inhibitors (3-MA), the results showed that LC3B protein expression was significantly reduced at 6 h (Supplementary Figs. 5, 11). Next, neutrophils were pre-treated with 10 µM 3-MA, an autophagy sequestration inhibitor, 10 µM HCQ, an autophagosome lysosome fusion inhibitor, or 10 µM Baf, an autophagosome lysosome fusion inhibitor, for 6 h. As LC3B accumulates when HCQ and Baf inhibit autophagy, we used p62 to assess the level of autophagy. All three inhibitors prevented neutrophil autophagy (Fig. 4b, Supplementary Fig. 9). Compared with the control group, 3-MA and HCQ treatment significantly reduced the dsDNA concentration, whereas Baf treatment did not reduce NET formation significantly (Fig. 4a, c). These results suggest that autophagy plays a role in spontaneous NETosis and spontaneous NETs may form through mTOR-independent pathways.

### Autophagy and NETosis in an in vivo model of OVA-induced asthma

**Figure 5:**
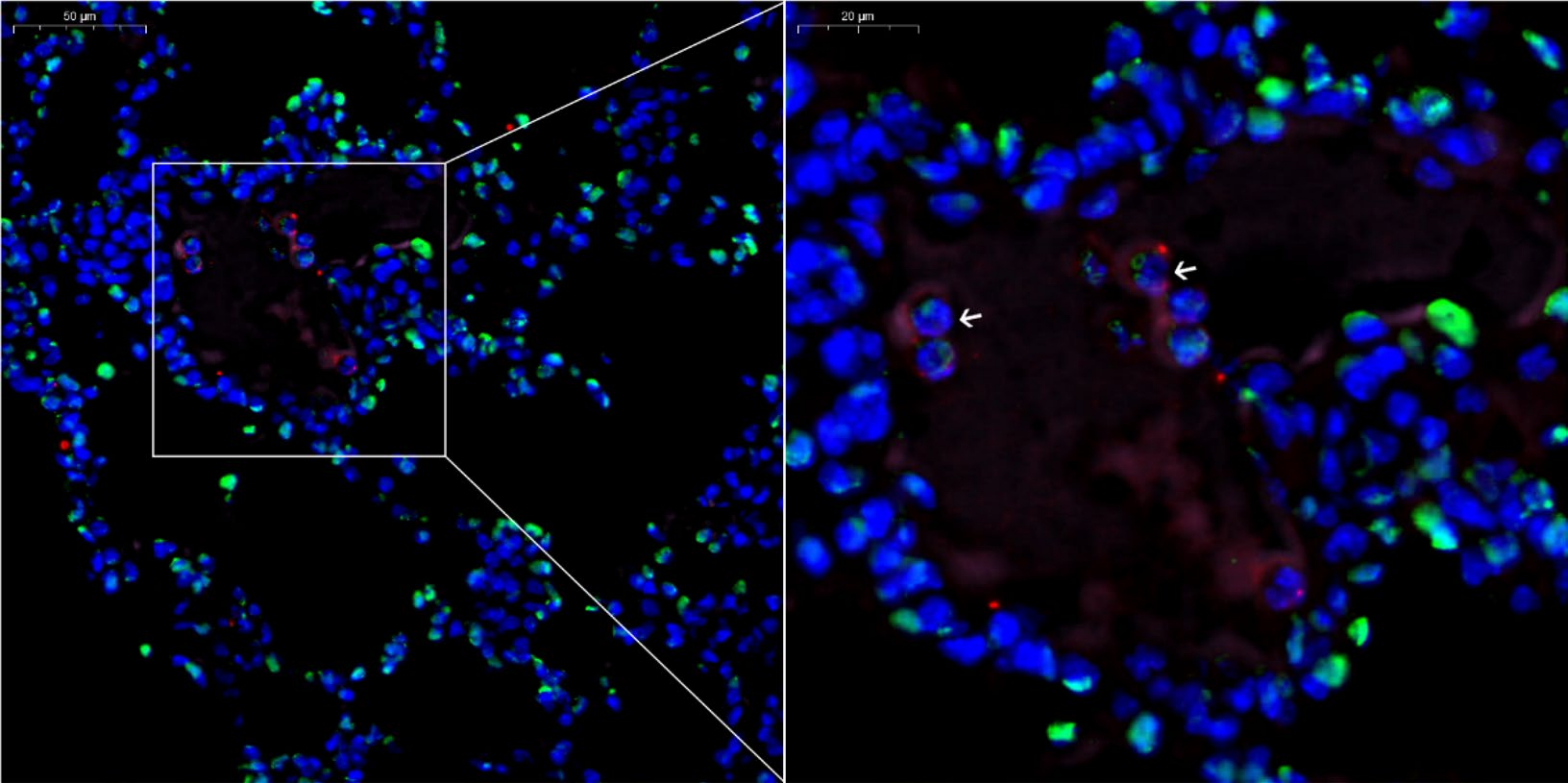
Autophagy and NETosis in an in vivo model of ovalbumin-induced asthma. The panel shows citH3, Ly6G, LC3B, and DNA co-localization; citrullinated histone H3 (citH3, green), light chain 3 B (LC3B, pink), Ly6G (marker of neutrophils, red), and DNA counterstained with DAPI (blue); original magnification was 40× and 80× ; scale bar, 50 and 20 μm.

We next investigated whether autophagy-mediated NETosis could be recapitulated in vivo in an OVA-induced asthma model. Hematoxylin and eosin-stained sections showed airway eosinophilic infiltration in the OVA-induced asthma model (Supplementary Fig. 6). We examined NET formation by evaluating the levels of Ly6G, citH3, and DNA. Autophagy activity was determined by measuring the autophagy-related protein LC3B. NETs were detected in OVA-induced asthma lung sections, whereas LC3B and citH3 proteins were co-localized with DNA (Fig. 5, Supplementary Fig. 7).

## Discussion

Web-like DNA structures complexed with immunogenic proteins, i.e. NETs, were demonstrated to activate plasmacytoid dendritic cells^29^ and autoreactive B cells^30^, as well as prime T lymphocytes^31^, finally resulting in autoantibody production. Thus, it is important to study the mechanism and triggering factors of spontaneous NETosis. In this study, we demonstrated that neutrophils can spontaneously release NETs.

The pathways of NET formation by neutrophils, known as NETosis, vary when stimulated by different pathogens. The cell death process depends on the generation of ROS by NADPH oxidase^15^. The citrullination of histone 3 by peptidylarginine deiminase type 4^32^ is the first described NETosis pathway^33^. Further, pyroptosis, a lytic cell death mechanism mediated by inflammatory caspases 4/11, was shown to play a role in NETosis^34^. Recently, type II programmed cell death, known as autophagy, was shown to be linked to NET formation in neutrophils^26^.

Neutrophils spontaneously release NETs under serum-free culture conditions^14^ and can spontaneously produce ROS simultaneously in culture^24^. Under serum-free culture conditions, ROS play a pivotal role in spontaneous NET formation^14^. However, the mechanism of spontaneous NET formation during cell culture in presence of serum is not well-understood. To explore the pathways underlying spontaneous NETosis during cell culture in presence of serum, differential RNA expression was elucidated. We analyzed RNA sequencing results in aging and control neutrophils and observed that several autophagy-related genes were differentially expressed. Furthermore, the autophagy level increased over time, and LC3B protein co-localized with citH3 and DNA. These results indicate that autophagy plays a role in spontaneous NETosis.

Autophagy, an adaptive catabolic process, can regulate several functions of neutrophils including neutrophil differentiation, neutrophil lifespan, apoptosis, degranulation, and NET formation^35^. Autophagy can regulate the release of NETs in multiple manners. Membrane nucleation, an important process in autophagy, can affect vesicle formation and thus, influence the formation of NETs^35,36^. Autophagy can also block NET formation by inhibiting oxidative respiratory burst and blocking histone citrullination^35,37^. Presence of the mTOR inhibitor rapamycin during neutrophil stimulation with PMA and formyl-Met-Leu-Phe significantly increased the formation of NETs^37^; however, the role of the mTOR signaling pathway in spontaneous NETs was unknown. In this study, through analysis of RNA sequencing data, we detected the expression of mTOR downstream proteins (EBP-1 and 70S6K, which are direct downstream substrates of mTOR signaling), which showed that the role of autophagy in spontaneous NETs may depend on the mTOR-independent pathway. Furthermore, we found that Tre treatment significantly increased NET formation, whereas Rap treatment did not increase the NET release by neutrophils. Our results suggest that spontaneous NETosis involves an mTOR-independent pathway, which must be validated in the future studies.

Neutrophil autophagy involves seven steps: signal induction, membrane nucleation, cargo targeting, vesicle expansion, autophagosome formation, fusion with the lysosome, cargo degradation, and nutrient recycling. Thus, we analyzed different stages of autophagy during the formation of spontaneous NETs using autophagy inhibitors. Compared with control neutrophils, 3-MA and HCQ treatment significantly reduced the percentage of NET-positive cells, whereas Baf treatment did not reduce NET formation significantly. 3-MA is an autophagy sequestration inhibitor that inhibits the formation of autophagosomes and is used as an early-stage autophagy inhibitor. In contrast, HCQ and Baf inhibit autophagosome-lysosome fusion and serve as late-stage autophagy inhibitors^11,18^. Interestingly, Baf and HCQ, despite belonging to the same class of autophagy inhibitors, exerted differential effects on inhibiting spontaneous NET release. A previous study showed that both Baf and HCQ can caused oxidative stress, mitochondrial depolarization, and caspase-dependent apoptotic death; however, HCQ additionally stabilizes lysosomal permeability^38^, which play a role in NET formation^39^. Our study outcomes suggest that autophagy inhibitors can reduce the spontaneous release of NETs; however, this phenomenon requires further analysis.

The relationship between autophagy and NETosis was supported by our results in an OVA-induced asthma model, in which LC3B-bearing NETs were observed in the lung tissues. Although autophagy is considered a mechanism of cellular self-protection, dysregulation of autophagy-related genes is related to several diseases and exerts effect via active NET release. During sepsis, the importance of autophagy in NET formation was confirmed using various autophagy inducers in vitro and in vivo, which showed that neutrophil autophagy can increase the sepsis survival rate by increasing NET formation^18^. In addition, the autophagy pathway participates in thromboinflammation and fibrosis in systemic lupus erythematosus via NET formation^11^. Autophagy also plays a critical role during fibrosis via autophagy-dependent NET release, which triggers human lung fibroblast activation and differentiation^40^.

In conclusion, our study provides important insight into the role of autophagy in aging-related spontaneous NETosis. Autophagy inducers contribute to spontaneous NETosis, whereas autophagy inhibitors inhibit the spontaneous release of NETs. Therefore, based on our study outcomes, we propose that autophagy is a marker of spontaneous NETosis.

## Supplementary Information

Supplementary Information.

## Data Availability

The datasets generated and/or analysed during the current study are not publicly available due data do not have consent from all patients to share their information online but are available from the corresponding author on reasonable request.
