# Supplementary material for: Spontaneous formation of neutrophil extracellular traps is associated with autophagy
Source: Sci Rep. 2021 Dec 14;11:24005. doi: 10.1038/s41598-021-03520-4 (PMC8671464; doi:10.1038/s41598-021-03520-4)
Supplement: Supplementary file 1 — Supplementary Information. [file 41598_2021_3520_MOESM1_ESM.docx]

Spontaneous formation of neutrophil extracellular traps is associated with autophagy

Yun Guo^1,2,#^, Fei Gao^3, #^, Xin Wang^2^, Zhenzhen Pan^1^, Qian Wang^1^, Shiyao Xu^1^, Shanshan Pan^1^, Ling Li^1^, Deyu Zhao^2^, Jun Qian^1^

^1^ Department of Respiratory Medicine, The Affiliated Wuxi Children's Hospital of Nanjing Medical University, Wuxi, China

^2^ Department of Respiratory Medicine, Children’s Hospital of Nanjing Medical University, Nanjing, China

^3^ Department of Intensive Care Unit, The Affiliated Wuxi People's Hospital of Nanjing Medical University, Wuxi, China

^#^ Yun Guo and Fei Gao contributed equally.

Correspondence authors:

Jun Qian MD

Department of Respiratory Medicine, The Affiliated Wuxi Children’s Hospital of Nanjing Medical University

Wuxi, Jiangsu, China, 214023

Emile: Wuxi_Qian@126.com; qian82101@aliyun.com

Deyu Zhao PhD

Department of Respiratory Medicine, Children’s Hospital of Nanjing Medical University

Nanjing, Jiangsu, China, 210000

Emile: [Zhaodeyu988@126.com](mailto:Zhaodeyu988@126.com)


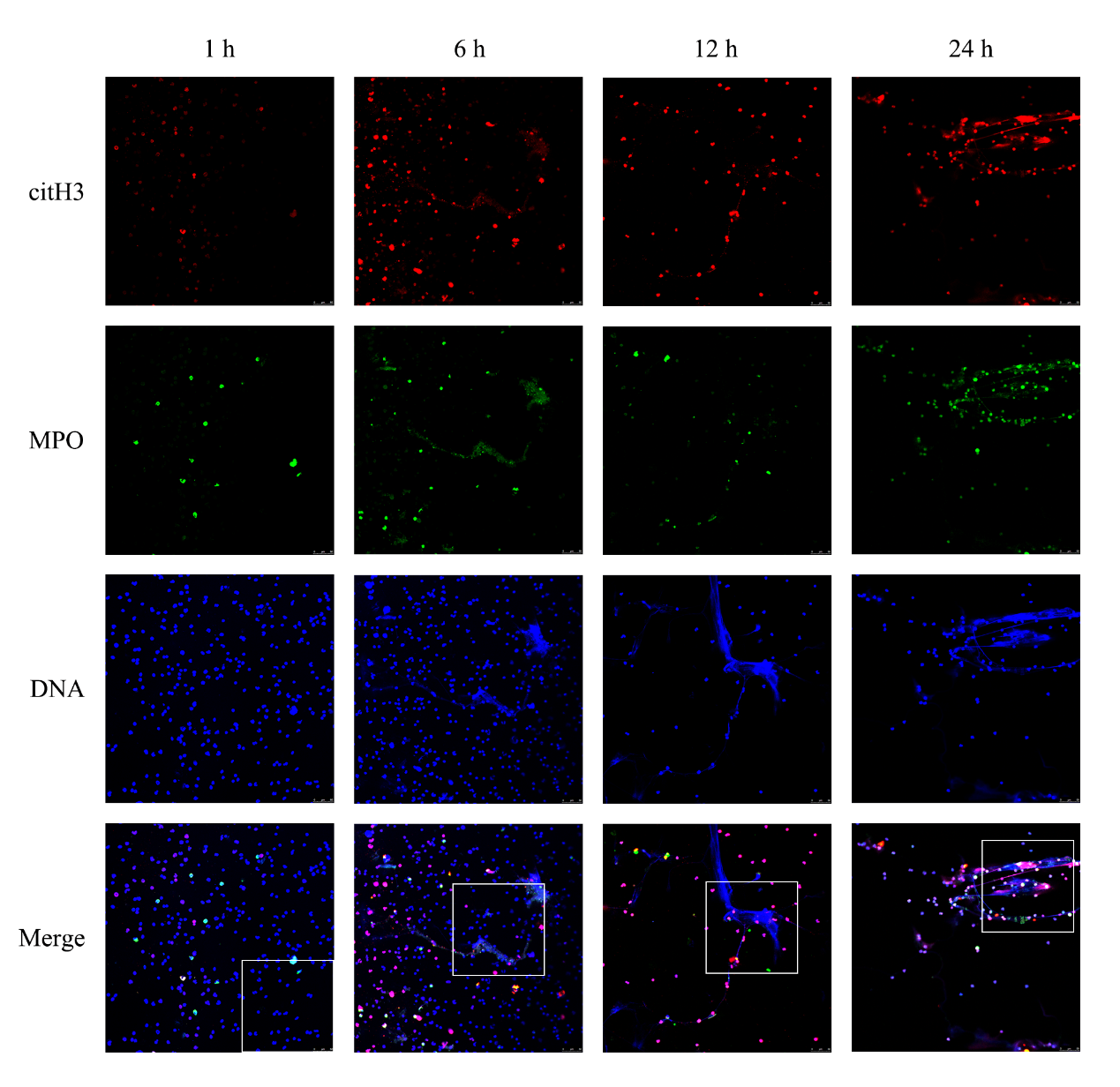


**Supplementary Figure 1.** Aging-related spontaneous NETosis. The proportion of spontaneous NETs was tested by fluorescence microscopy at 1, 6, 12, and 24 h. Samples were stained with antibodies against citrullinated histone H3 (citH3, red) and myeloperoxidase (MPO, green) and the DNA was counterstained with DAPI (blue), original magnification 40 ×, scale bar – 50 μm.


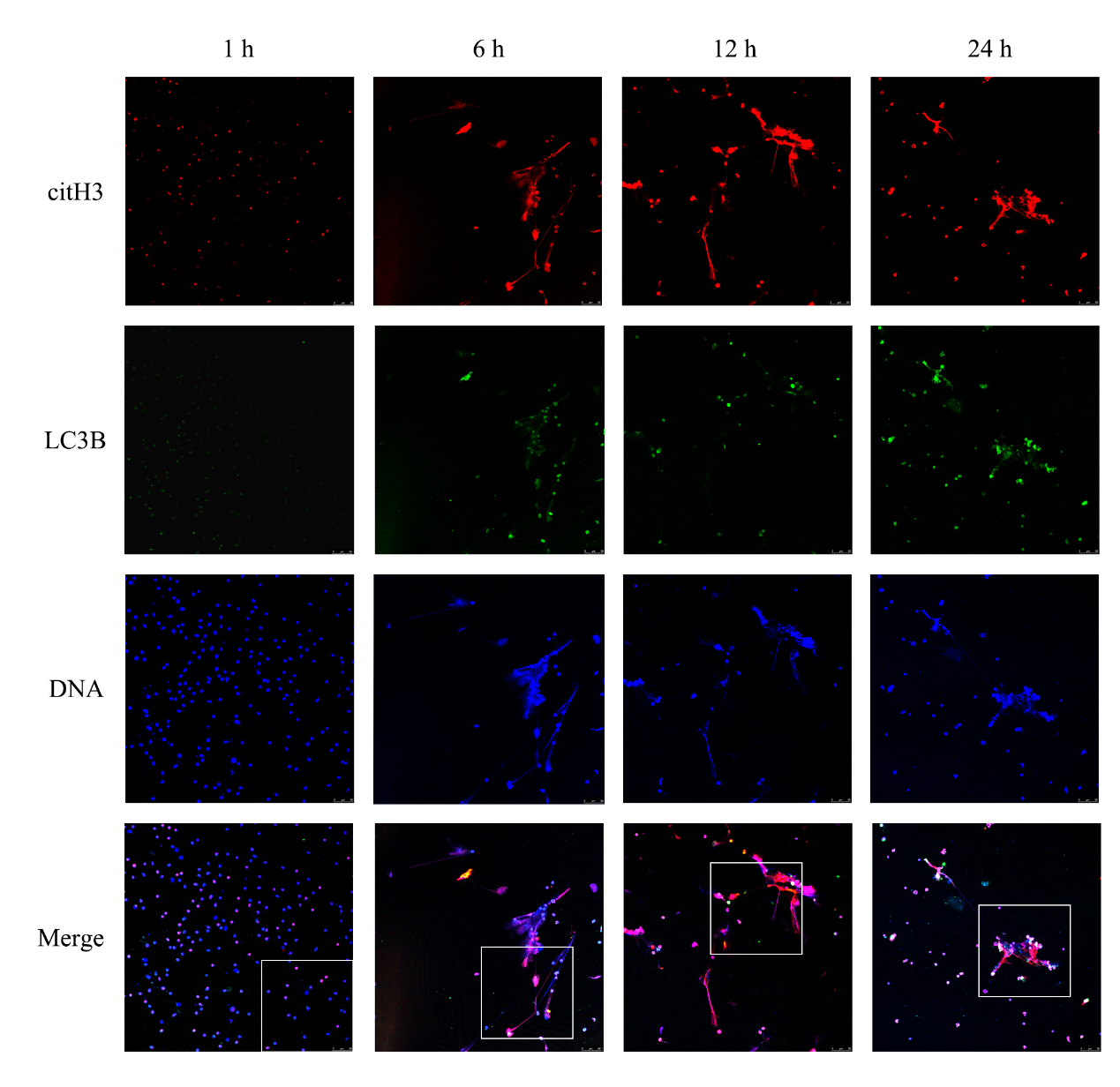
**Supplementary Figure 2.** Autophagy in spontaneous NETosis. The percentage of cells exhibiting active autophagy were detected by LC3B expression via immunofluorescence after 1, 6, 12, and 24 h of incubation. Samples were stained with antibodies against citrullinated histone H3 (citH3, red) and light chain 3 B (LC3B, green), and the DNA was counterstained with DAPI (blue), original magnification 20 ×, scale bar – 50 μm.


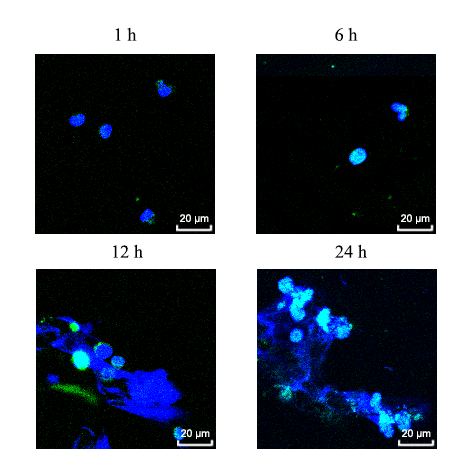


**Supplementary Figure 3.** Autophagy induction assessed with LC3B staining (confocal microscopy; green: LC3B, blue: DAPI) in neutrophils after 1, 6, 12, and 24 h of incubation.


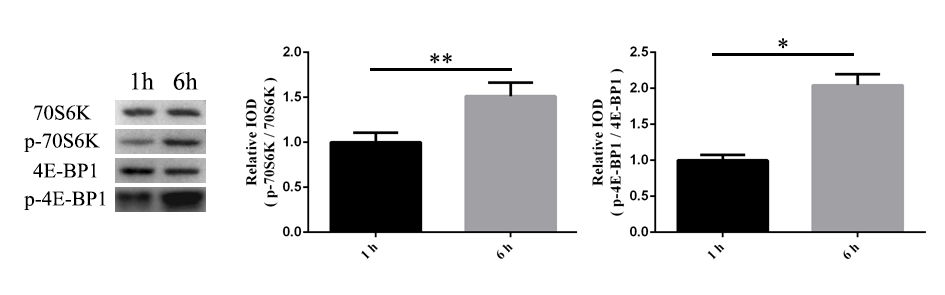


**Supplementary Figure 4.** Immunoblotting analysis of the mTOR pathways in spontaneous NETosis. Integrated optical density (IOD) of p-70S6K/70S6K and p-4E-BP1/4E-BP1 relative to 1h, data are presented as the mean±SD of three independent experiments *P < 0.05; **P < 0.01.


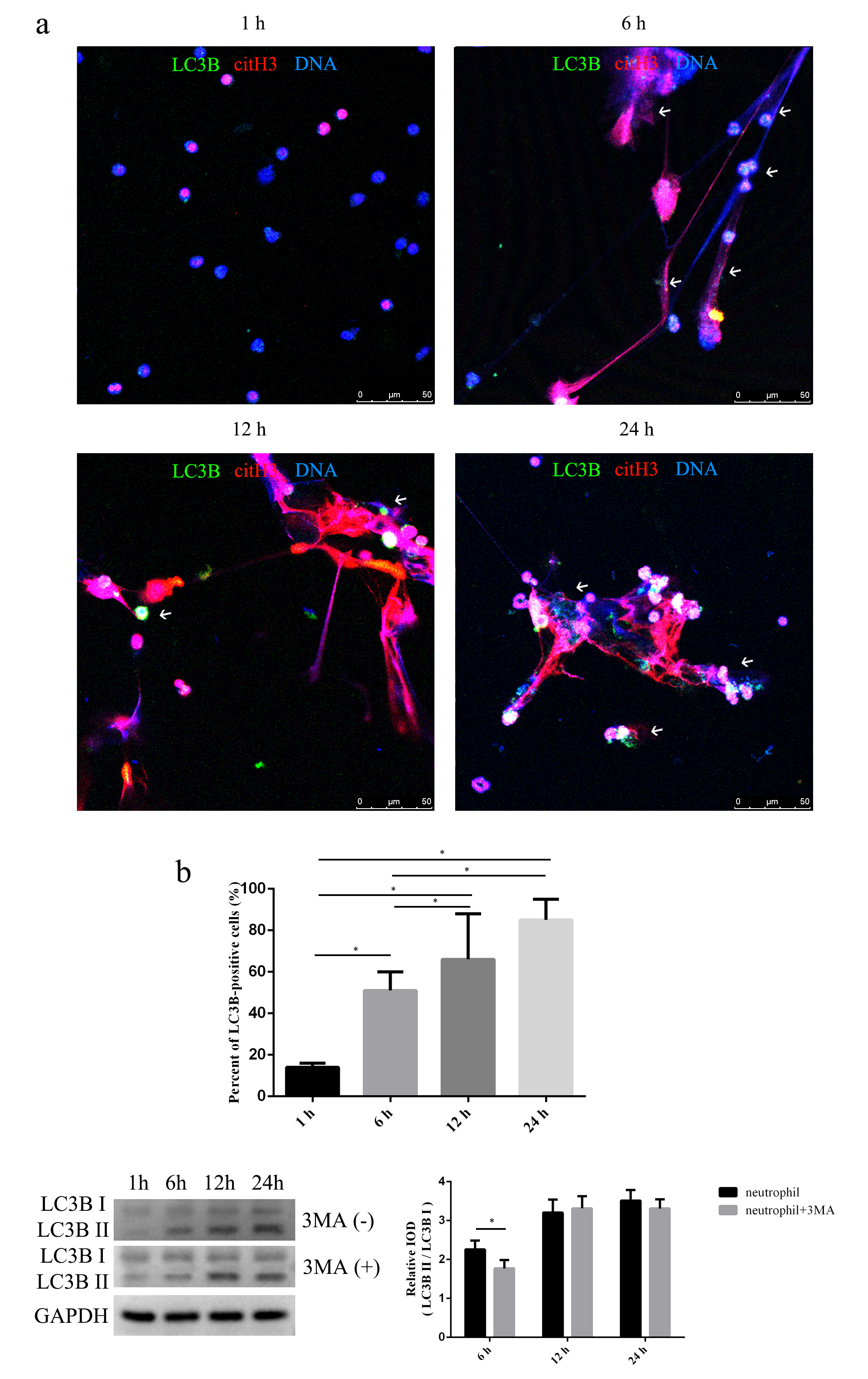


**Supplementary Figure 5.** Immunoblotting analysis of the effect of autophagy inhibitor in spontaneous NETosis. Integrated optical density (IOD) of LC3B II/ LC3B I in the presence and absence of autophagy inhibitors (3-MA), data are presented as the mean±SD of three independent experiments *P < 0.05.


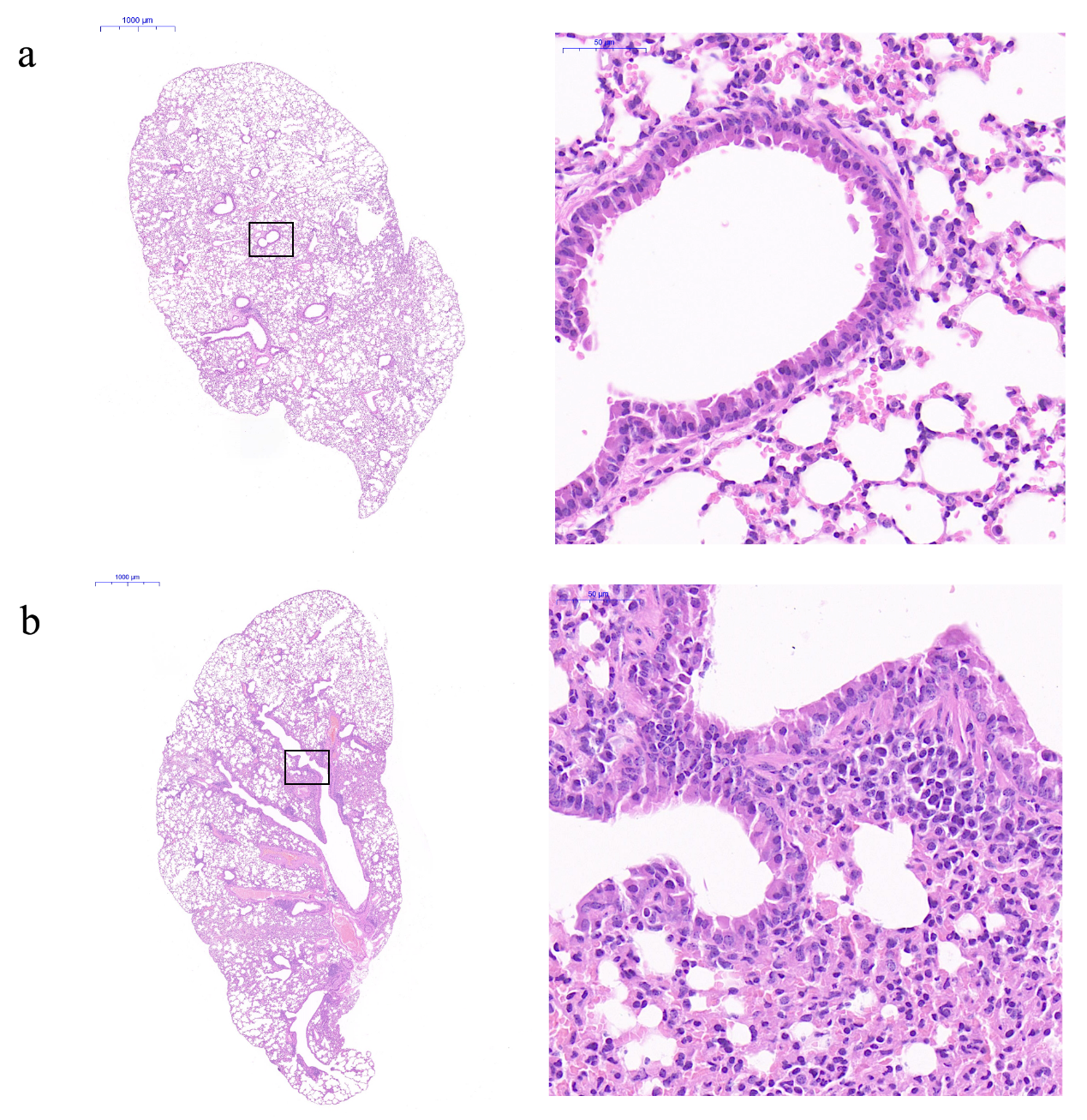


**Supplementary Figure 6.** Model of ovalbumin (OVA)-induced asthma. Hematoxylin and eosin-stained sections in the control group (a) and OVA-induced asthma group (b). Airway eosinophilic infiltration in OVA-induced asthma model; scale bar on the left is 1000 μm; original magnification on the right is 40×; scale bar, 50 μm.


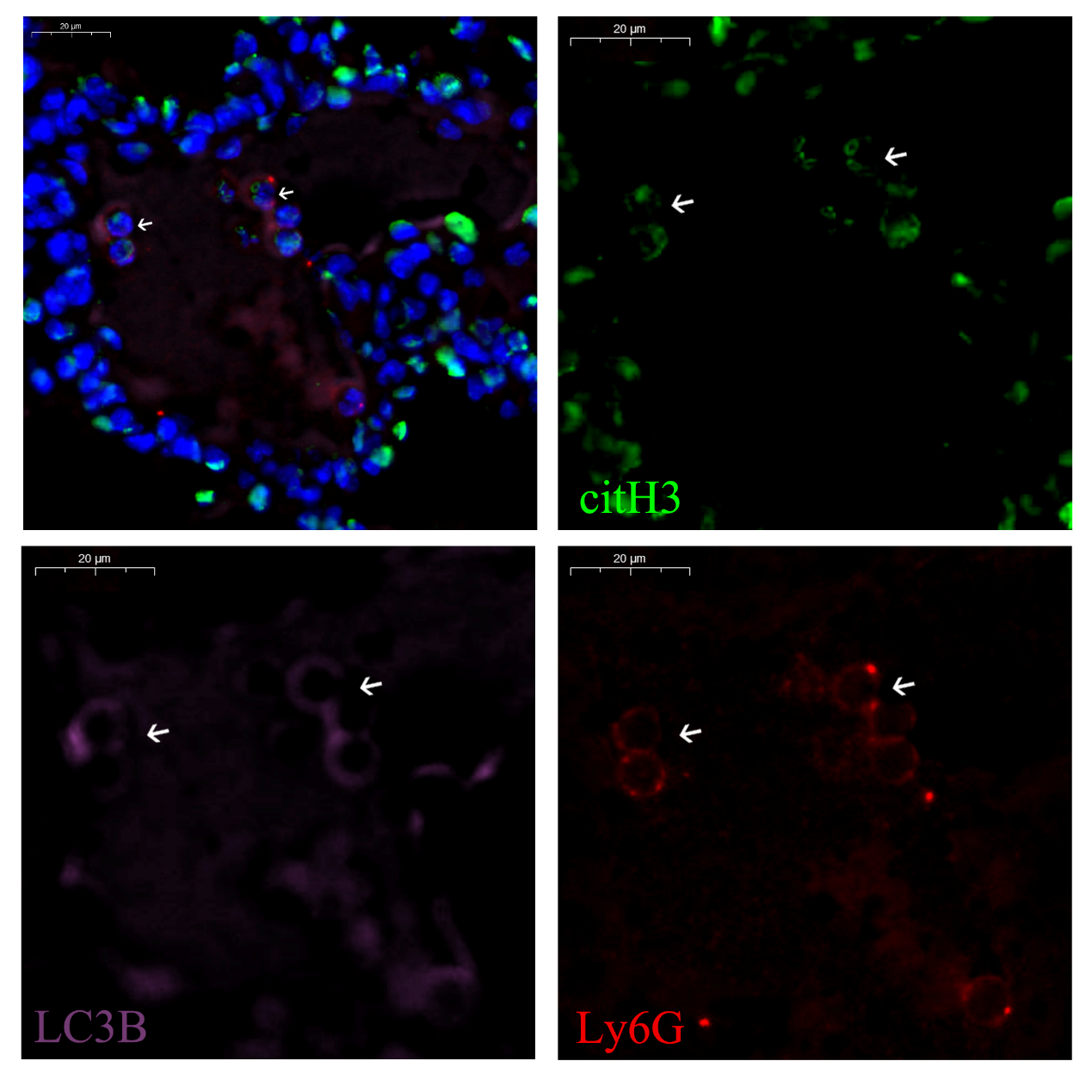


**Supplementary Figure 7.** Autophagy and NETosis in an *in vivo* model of OVA-induced asthma. The panel shows citH3, Ly6G, LC3B, and DNA co-localization; citrullinated histone H3 (citH3, green), light chain 3 B (LC3B, pink), Ly6G (a marker of neutrophil, red), and DNA counterstained with DAPI (blue); original magnification was 80 × and 120 ×, scale bar – 20 μm.


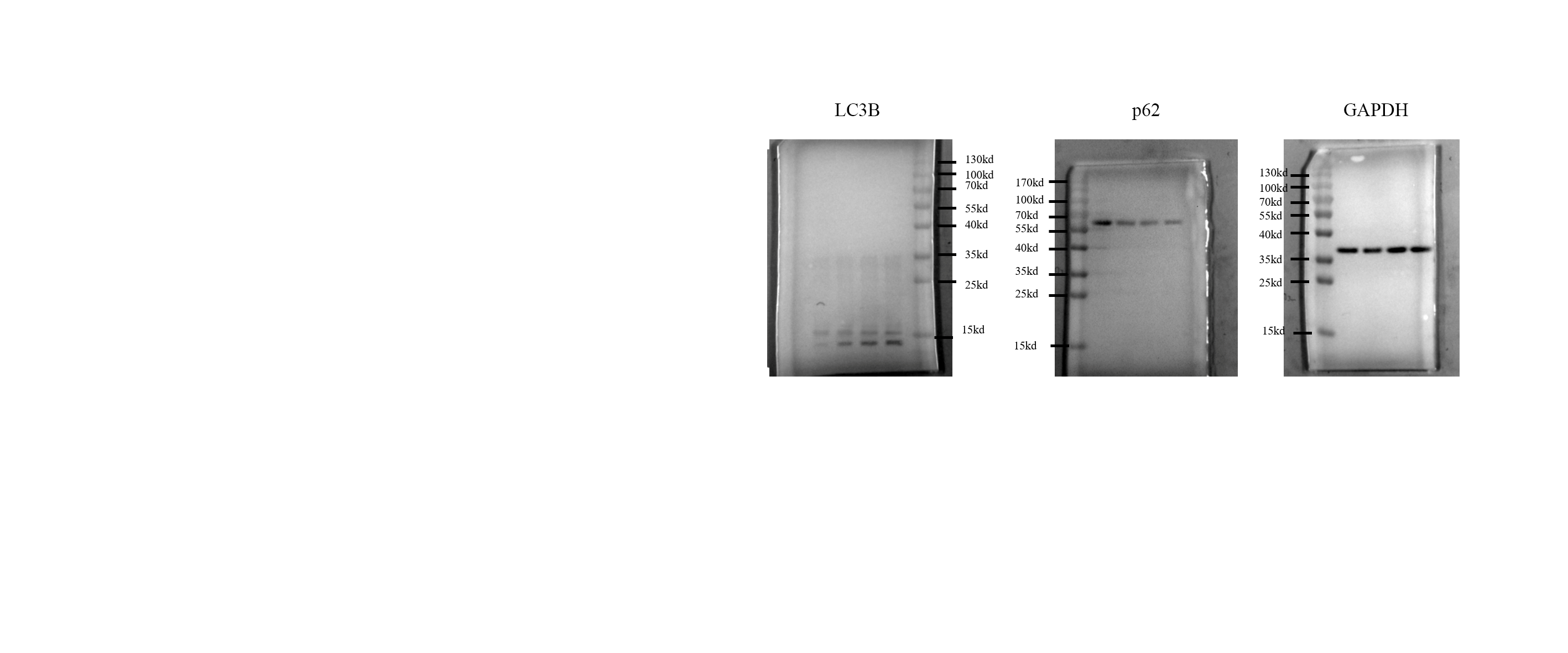


**Supplementary Figure 8.** Full length gels and blots with membrane edges visible of autophagy in spontaneous NETosis.


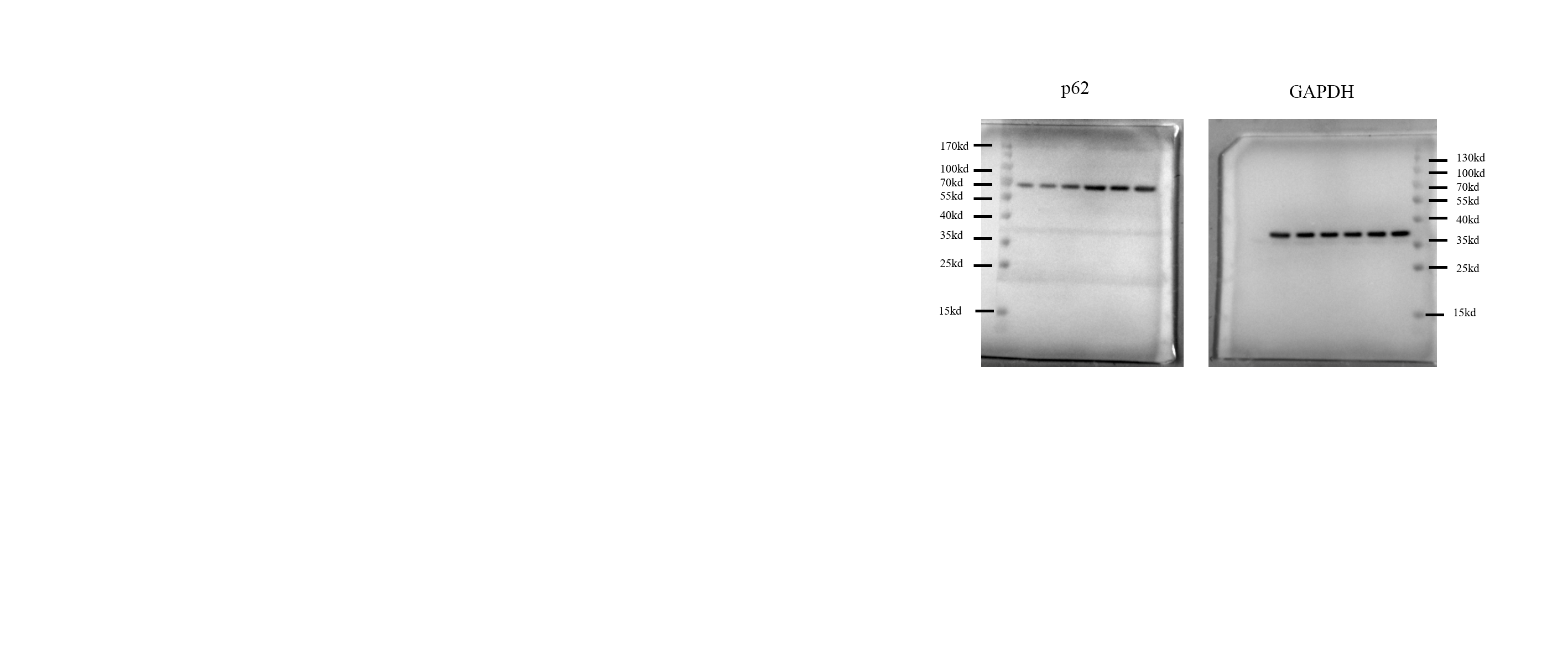


**Supplementary Figure 9.** Full length gels and blots with membrane edges visible of activation of neutrophil autophagy enhances spontaneous NETosis.


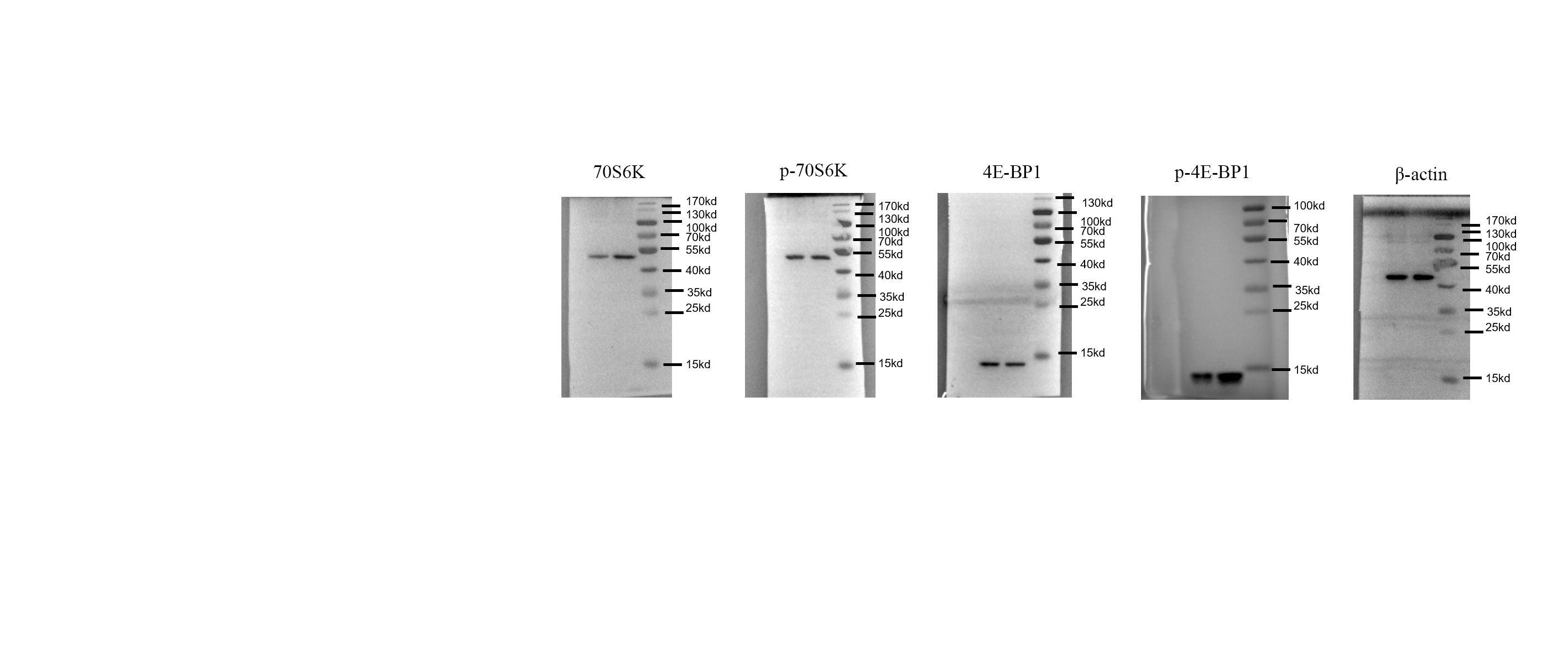


**Supplementary Figure 10.** Full length gels and blots with membrane edges visible of the mTOR pathways in spontaneous NETosis.


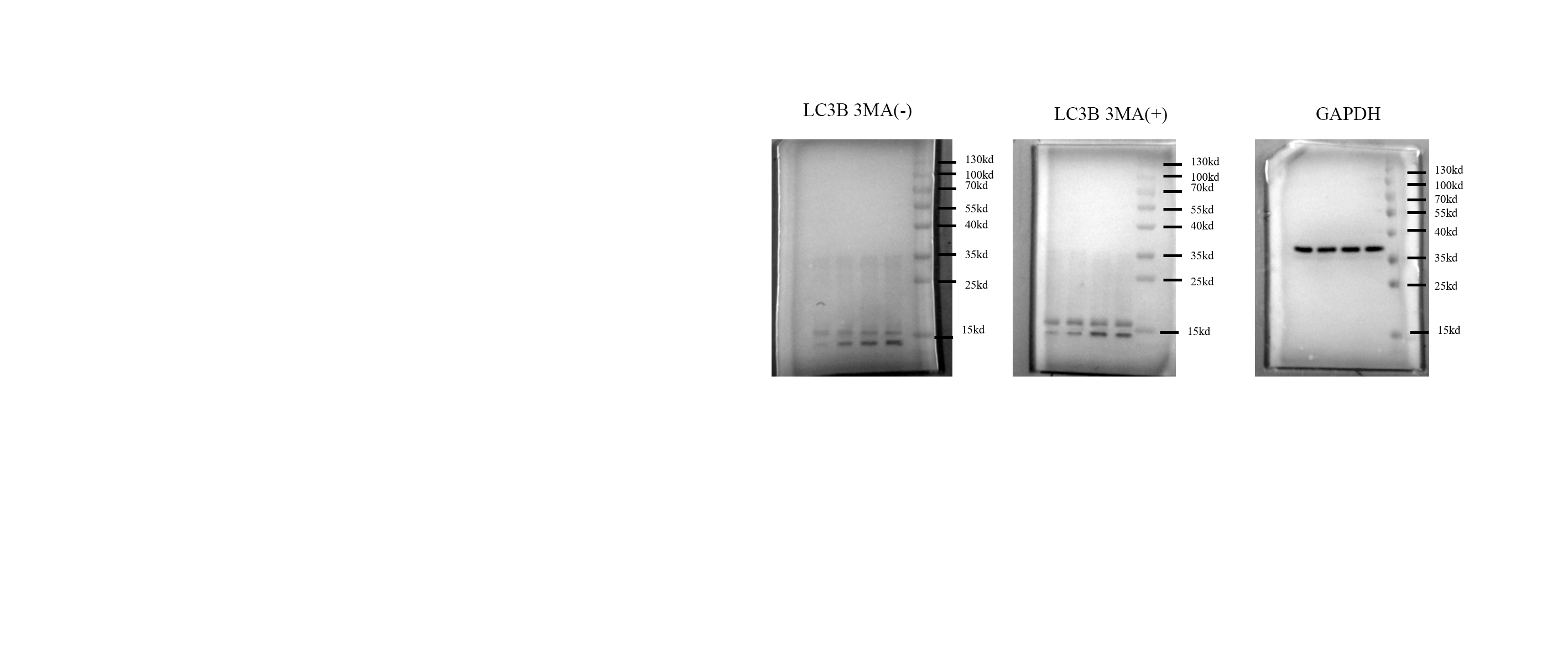


**Supplementary Figure 11.** Full length gels and blots with membrane edges visible of the effect of autophagy inhibitor in spontaneous NETosis.
